# Long Non-coding RNA NEAT1 Alleviates Acute-on-Chronic Liver Failure Through Blocking TRAF6 Mediated Inflammatory Response

**DOI:** 10.3389/fphys.2019.01503

**Published:** 2019-12-12

**Authors:** Yumin Xu, Zhujun Cao, Yezhou Ding, Ziqiang Li, Xiaogang Xiang, Rongtao Lai, Zike Sheng, Yuhan Liu, Wei Cai, Ronggui Hu, Hui Wang, Qing Xie

**Affiliations:** ^1^Department of Infectious Diseases, Ruijin Hospital, School of Medicine, Shanghai Jiao Tong University, Shanghai, China; ^2^Key Laboratory of Systems Biology, CAS Center for Excellence in Molecular Cell Signaling Network, Shanghai Institute of Biochemistry and Cell Biology, Chinese Academy of Sciences, Shanghai, China

**Keywords:** NEAT1, LPS, acute on liver failure, STAT1, p38, TRAF6

## Abstract

**Background:**

Long non-coding RNAs (lncRNAs) have recently been tightly linked to plenty of human diseases. However, knowledge of acute-on-chronic liver failure (ACLF) related lncRNAs remains insufficient. In this work, we studied the role of the lncRNA nuclear enriched abundant transcript 1 (NEAT1) in the pathogenesis of ACLF.

**Methods:**

ACLF model was established by challenging D-galactosamine (D-GalN)/ lipopolysaccharide (LPS) i.p. in rats with cirrhosis. The serum levels of IL-1, IL-6, and HMGB1 were determined using ELISA. Quantitative real time-PCR and western blot were performed to evaluate RNA and protein levels of inflammatory response. RNA immunoprecipitation assay was performed to confirm protein that interacts with NEAT1.

**Findings:**

Over-expression of NEAT1 could interact with TRAF6 and decrease its ubiquitination level, and significantly reduced the expression levels of IL-6, IL-22. Importantly, in ACLF rat model, NEAT1 over-expression reduced several cytokines expression and alleviated the pathological status in contrast to the control group. Additionally, NEAT1 was increased and positively correlated with IL-22 and IL-6 levels in PBMCs from the ACLF patients.

**Interpretation:**

NEAT1 can suppress inflammatory response through blockade of TRAF6 ubiquitination in ACLF rat model, suggesting that lncRNA NEAT1 might play protective roles in the pathogenesis of ACLF and provide promising novel target for pharmacological intervention.

## Introduction

Liver failure is a common syndrome of severe liver diseases, which causes serious damage to life and health of people. Massive apoptosis and necrosis of hepatocytes occur during the development of liver failure (Cao et al., 2015, 2019). Acute-on-chronic liver failure (ACLF) is a fatal clinical syndrome of acute decompensated liver function, which develops on the basis of chronic liver disease and is stimulated by various inducements in a short term (Bajaj et al., 2019). The patients were characterized by acute liver damage such as jaundice and blood coagulation dysfunction followed by sequential organ failure (Olson, 2019; Yoon et al., 2019). ACLF is very common clinically with high mortality and morbidity, and is the main component of liver failure. More than 80% of patients with ACLF are caused by hepatitis B virus (HBV) infection in China. Although nucleoside analog therapy can effectively inhibit HBV replication, it can not improve the 3-month survival rate of HBV-related ACLF patients (Arroyo, 2019). So far, liver transplantation remains the only viable treatment for such patients. The specific pathogenesis of ACLF is still unclear. It is urgent to carry out in-depth research to block the progression of disease, improve the survival rate of patients, and provide new countermeasures for clinical treatment.

Long non-coding RNAs (lncRNAs) are greater than 200 nucleotides in length (Prinz et al., 2019). LncRNAs are pivotal regulators of gene functions and various cellular processes (Wang et al., 2018, 2019; Yamazaki et al., 2018). The lncRNAs have been found to be involved in subcellular architecture, protein complex stabilization, as well as in physiology and pathophysiology (Del Vecchio et al., 2018; Gutschner et al., 2018; Liu et al., 2018; Wang et al., 2019; Yan et al., 2019; Yu et al., 2019). Within the last few years, numerous studies have shown that nuclear enriched abundant transcript 1 (NEAT1) play crucial roles both in carcinogenesis (Klec et al., 2019) and non-cancerous diseases such as neurodegeneration and inflammation (Prinz et al., 2019).

NEAT1 is discovered in 2007 and abundant in several organs including prostate, ovary, pancreas, and colon (Hutchinson et al., 2007). There are two NEAT1 isoforms, the short 3.7 kb (NEAT1_1) and a long 23 kb version (NEAT1_2) (Sasaki et al., 2009). The long-isoform NEAT1_2 can form a triple helix structure and is relatively stable. The NEAT1_1 is isoformed from NEAT1_2 and polyadenylated. NEAT1 is enriched in the nucleus and indispensable for the formation and maintenance of paraspeckles (Mao et al., 2011; Naganuma et al., 2012). *NEAT1* knockout results in paraspeckles destruction, and overexpression of NEAT1 leads to paraspeckles accumulation. Paraspeckles were recognized as nuclear mRNA anchors. In terms of cancer biology, NEAT1 mainly functions as competing endogenous RNA (ceRNA) by sponging suppressive miRNAs (Bartel, 2009; Qi et al., 2015). Subsequently, these miRNAs lose the ability to function as a tumor suppressor and oncogenic mRNAs are translated, ultimately contributing to carcinogenesis. NEAT1 is also a key player in immune system response (Carpenter et al., 2013; Prinz et al., 2019; Zhang et al., 2019). NEAT1 exerts different consequences depending on different downstream mechanisms.

Endotoxin and LPS are important regulators in the ACLF process (Xu et al., 2013). To investigate the function of lncRNAs in inflammation and ACLF, we profiled the differential expressed lnRNAs upon LPS treatment in HepG2 cells using high throughput sequencing in our previous work. In this study, we found that the expression level of NEAT1 was up-regulated upon LPS treatment in HepG2 cells. The function and mechanism of NEAT1 in ACLF were studied.

## Materials and Methods

### Reagents

Human serum albumin (HSA) was obtained from Baxter (Deerfield, IL, United States). LPS, D-galactosamine, ALT kit, and AST kit were purchased from Sigma-Aldrich (St Louis, MO, United States). IL-6, IL-22, HMGB1 ELISA kits were purchased from BIKW Co., Ltd. (Beijing, China). Antibodies against Ubiquitin (Cat.3936), TRAF6 (Cat.8028), p38 (Cat.9212), p-p38 (Cat.9216), p65 (Cat.8242), p-p65 (Cat.3033), JNK (Cat.9252), p-JNK (Cat.4668), STAT1 (Cat.14995), and Actin (Cat.3700) were obtained from Cell Signaling Technology (United States). The magnetic RNA-Protein Pull-Down Kit was purchased from Thermo Fisher (United States). Real-time PCR kits were from Takara (Japan). NEAT1 lentivirus, sh-NEAT1 lentivirus, AAV8, and AAV8-NEAT1 were purchased from Genechem (China).

### Establishment of ACLF in Rats

SPF Wistar rats (250–300 g) were purchased from Shanghai Laboratory Animal Center (Shanghai, China). These animals were bred and housed in standard cages in a climate-controlled room (22 ± 1°C and 50 ± 5% humidity) with 12-h light-dark cycles for 7 days before experiments. All experiments were performed according to the Association for Assessment and Accreditation of Laboratory Animal Care guidelines^1^. ACLF model was established as previously described with minor modifications (An et al., 2012; Xu et al., 2013). These rats were administrated with repeated injection of Freund’s adjuvant containing human serum albumin (HSA) at the dosage of 25 mg per kilogram subcutaneously at days 0, day 14, day 24, and day 34. Ten days after the last injection, the concentration of serum HSA from these immunized rats was detected to confirm the sensitized status. After that, these sensitized rats were injected with HSA intravenously twice a week for 6 weeks. The first dose of HSA was 2.5 mg/rat, and the second dose was 3.0 mg/rat in the first week. In the next week, these rats were injected with HSA intravenously at doses of 3.5 mg/rat and 4.0 mg/rat. For the remaining 4 weeks, the dose was up to 4.5 mg/rat. Finally, the rats were injected intravenously with D-galactosamine at a dosage of 400 mg per kilogram and lipopolysaccharide at a dosage of 400 mg per kilogram to establish the ACLF animal model. Control mice were administered with equivalent volumes of saline. All rats were grouped as follows: the control group, the ACLF plus tail vein injection of AAV8 (5 × 10^9^ pfu/mouse), and the ACLF plus tail vein injection of AAV8-NEAT1 (5 × 10^9^ pfu/mouse) group. Each group contained six rats. 3 days after the AAV injection, the liver function analysis was performed. 7 days after the AAV injection all rats were sacrificed using Barbital. The liver tissues were used for Hematoxylin and Eosin staining. The scheme of experiments was illustrated as follows.

**SCHEME 1:**
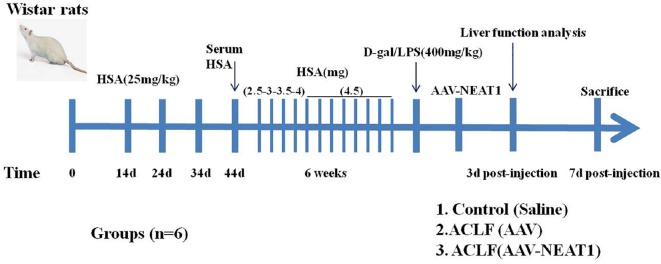
Establishment of ACLF in rats.

### Cell Culture

HepG2 and Raw264.7 cells were cultured in DMEM supplemented with 10% FBS in a 5% CO_2_ atmosphere at 37°C. Primary human hepatocytes cells were obtained from Abace-Bio company and cultured in 1640 supplemented with 10% FBS. HepG2 cells were plated in 24-well plates (5 × 10^5^ cells per well) 2 h before the LPS stimulation. The cells were treated for 3, 6, 9, and 12 h with different stimuli: 250, 500, and 1000 ng/ml LPS.

### RNA Isolation and RT-PCR Analysis

The mRNA expression after LPS and indicated treatment was detected by real time PCR. Total RNA was extracted from cells using RNA isolation plus kit, according to the manufacturer’s instructions. PCR product formation was monitored continuously using ABI 7500 software. Primer sequences for real time PCR were as follows: IL-6 (human), 5′-CCAGCTATGAACTCCTTCTC-3′ (sense), 5′-GCTTGTTCC TCACATCTCTC-3′ (antisense); IL-22 (human), 5′-GCAGGCTT GACAAGTCCAACT-3′ (sense), 5′-GCCTCCTTAGCCAGCAT GAA-3′ (anti-sense); GAPDH, 5′-CACATGGCCTCCAAGGAG TAA-3′ (sense), 5′-TGAGGGTCTCTCTCTTCCTCTTGT-3′ (anti-sense); NEAT1 (human), 5′-TGGCTAGCTCAGGGCTT CAG-3′ (sense), 5′-TCTCCTTGCCAAGCTTCCTTC-3′ (anti-sense); IL-6 (Rat), 5′-TGATGGATGCTT CCAAACTG-3′ (sense), 5′-GAGCATTGGAAGTGGGGTA-3′ (anti-sense); IL-22 (Rat), 5′- CTACACTCCCACCGTTGATG-3′ (sense), 5′-CCT CCCTTACCAAAGAGCTG-3′ (anti-sense); NEAT1 (Rat), 5′-TCGAGTAATAGCTTGGGAACTAAATATGTGCTTTATTT AGTTCCCAAGCTATTAG-3′ (sense), 5′-AATTCTAATAGCTT GGAACTAAATAAAGCACATATTTAGTTCCCAAGCTATTAC-3′ (anti-sense); GAPDH (Rat), 5′-GTATTGGGCGCCTGGT CACC-3′ (sense), 5′-CGCTCCTGGAAGATGGTGATGGT-3′ (anti-sense). Relative gene expression was normalized to GAPDH, and fold change was calculated using the ΔΔCt method.

### RNA Pull-Down Assay

Biotin RNA Labeling and *in vitro* transcription were performed to obtain the biotin-labeled RNAs. The biotinylated NEAT1, antisense NEAT1, was incubated with cell lysates (with RNAse inhibitor) overnight at 4°C. Streptavidin beads were used to purify the interacting complexes for 3 h at room temperature, followed by immunoblotting using specific antibody to TRAF6.

### RNA Immunoprecipitation

Cells were treated with 0.3% formaldehyde for 10 min at 37°C. Then the sample was incubated with 0.125 M glycine for 5 min at RT. Then cells were washed with PBS and centrifuged at 1500 rpm for 2 min, then the pellet was resuspended in RIPA buffer (1 mM cocktail, 0.1% SDS, 0.5 mM DTT, 50 mM Tris, pH 7.4, 0.5% sodium deoxycholate, 150 mM NaCl, and 1 mM EDTA). The cell lysate was incubated on ice for thirty minutes with interval vortex. Antibodies against TRAF6 or IgG control were incubated overnight with the cell lysate at 4°C. Protein G dynabeads were used to recover the RNA/protein complex, followed by washing with RIPA buffer several times. Finally the RNA was isolated with Trizol and quantified by real time PCR.

### Western Blot

Cell lysates and pull down samples were denatured and loaded to 10% SDS-PAGE. After that, the proteins were transfered onto PVDF membranes, followed by milk blocking. These primary antibodies (anti-P38, anti-JNK, anti-P65, and anti-TRAF6) were diluted at 1:1000 and incubated with the membrane for 1 h at room temperature. The corresponding HRP-conjugated secondary antibodies were used to detect the primary antibody.

### The Liver Function Assay

The serum concentrations of interleukin-6, interleukin-22 and HMGB1 were analyzed using the commercially ELISA kits. Serum ALT and AST were determined according to the manufacturer’s instructions.

### siRNA

Negative control and small interfering RNA targeting STAT1 were obtained from GenePharma (Shanghai, China). The detail sequences were as follows, STAT1: 5′ GCUGAACUAUAACU UGAAA 3′; Control, 5′ UUCUCCGAACGUGUCACGU3 ′.

### Hematoxylin and Eosin Staining and Histopathology Scoring

Liver tissues were fixed in 10% formalin-fixed and embedded in paraffin after sacrificing. For routine histological analysis, 5 μm sections were cut and stained with hematoxylin and eosin. Histopathology was scored under a light microscope based on the criteria as previously reported (Xu et al., 2013).

### Patients

Forty six ACLF patients were recruited from the Department of Infectious Diseases, Ruijin hospital, Shanghai Jiao Tong University School of Medicine. Thirty age and gender matched people were selected as healthy controls. The demographic and clinical characteristics of the enrolled subjects were listed in Table 1. CLIF Consortium ACLF score (CLIF-C ACLFs) (Jalan et al., 2014) was calculated as described. CLIF-C ACLFs = 10 × [0.33 × CLIF-OFs + 0.04 × Age + 0.63 × ln (WBC count)-2].

**TABLE 1:** Clinical characteristics of the participants enrolled in the study.

**Group**	**HCs**	**ACLF**
Case	30	46
Gender (M/F)	18/12	36/10
Age (years)	46.5 ± 2.8	44.9 ± 2.4
ALT level (U/L)	26.8 ± 1.9	318.8 ± 57.4
AST level (U/L)	23.6 ± 0.7	273.1 ± 44.3
TBiL level (μmol/L)	ND	320 ± 26.5
PTA (%)	ND	31.2 ± 3.3
Log [HBV DNA (IU/mL)]	ND	4.7 ± 1.7
HBsAg positive	0	26
HBeAg positive	0	19

*Data was shown as mean ± SEM; abbreviations: HCs, healthy controls; ALT, alanine aminotransferase; TBiL, total bilirubin; PTA, prothrombin time activity; HBsAg, hepatitis B surface antigen; HBeAg, hepatitis B envelope antigen; M, male; F, female; ND, not determined.*

### Statistical Analysis

Each experiment was carried out with triplicate samples. Generally, the experiments were repeated three times. Data are presented as mean ± SE. The *t*-test was used to compare difference between two groups using GraphPad (GraphPad, San Diego). *p* < 0.05 means statistically significant. ^∗^ means *p* < 0.05, and ^∗∗^ means *p* < 0.01.

### Ethics Statement

All subjects gave written informed consent in accordance with the Declaration of Helsinki.

## Results

### NEAT1 Is Induced by LPS in HepG2 Cells

**FIGURE 1:**
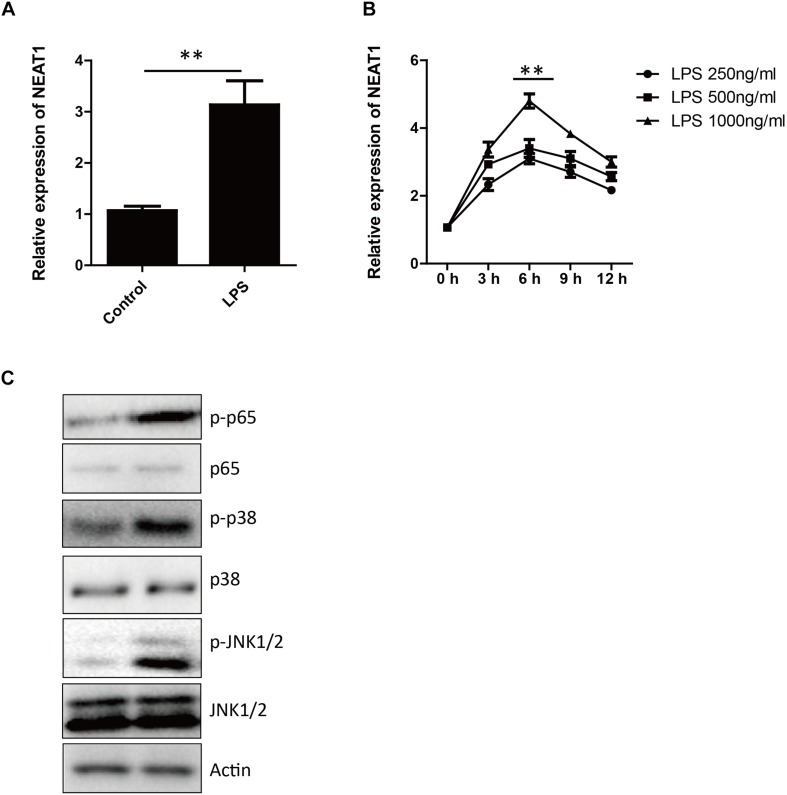
The expression of NEAT1 is induced by LPS. **(A)** The expression of NEAT1 in HepG2 cells is evaluated by qPCR 6 h after LPS (1000 ng/ml) treatment. Bars indicates mean ± SEM of three experiments. Two-tailed unpaired *t*-test is performed. ^∗∗^ *p* < 0.01. **(B)** The kinetics of NEAT1 expression in response to different concentrations of LPS for 3, 6, 9, and 12 h in HepG2 cells. qPCR is used and bars indicates mean ± SEM from three experiments. **(C)** Western blotting analysis of JNK1/2, p38, and p65 3 h after LPS treatment.

The expression level of NEAT1 in HepG2 cells increased in time and dosage dependent manner after LPS treatment (Figure 1A), which peaked at 6 h at 1000 ng/ml (Figure 1B). As previous reported, the LPS treatment induce the phosphorylation and activation of p38, p65, and JNK (Figure 1C). There are similar results in human primary hepatocytes and macrophages (Supplementary Figures S1, S2).

### Upstream Mediators of NEAT1 Expression in HepG2 Cells

**FIGURE 2:**
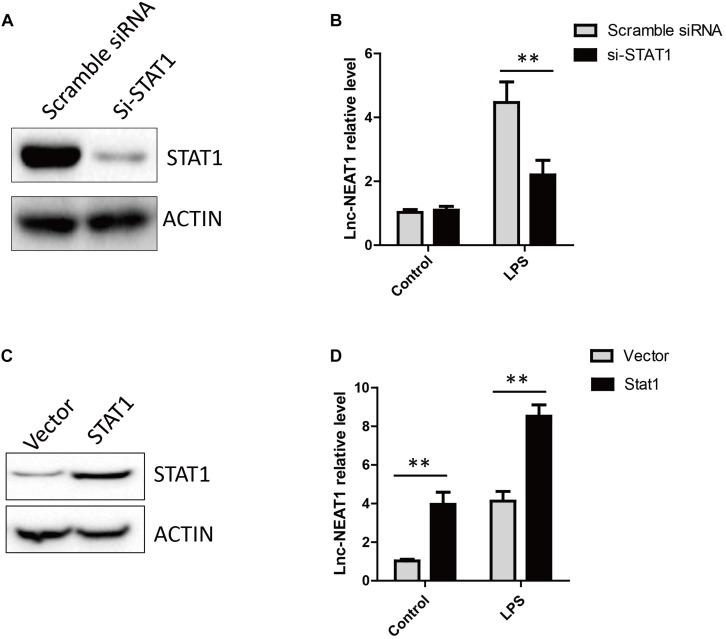
LPS induced NEAT1 expression is dependent on STAT1. **(A)** The knockdown efficiency for STAT1 siRNA. **(B)** Knockdown of STAT1 decreases NEAT1 expression upon LPS treatment in cultured HepG2 cells. **(C)** Overexpression of STAT1 in HepG2. **(D)** Overexpression of STAT1 increases NEAT1 expression in cultured HepG2 cells. Data is represented as mean ± SEM of three experiments. Two-tailed unpaired *t*-test is performed. Ns means none significant, and ^∗∗^ means *p* < 0.01.

We found signal transducer and activator of transcription 1 (STAT1) could potentially bind to promoter of NEAT1 by *in silico* bioinformatic analysis. STAT1 knockdown completely abrogated LPS-stimulated NEAT1 expression in HepG2 cells (Figures 2A,B), whereas STAT1 over-expression showed an opposite effect (Figures 2C,D).

### NEAT1 Affects Auto-Ubiquitination of TRAF6

**FIGURE 3:**
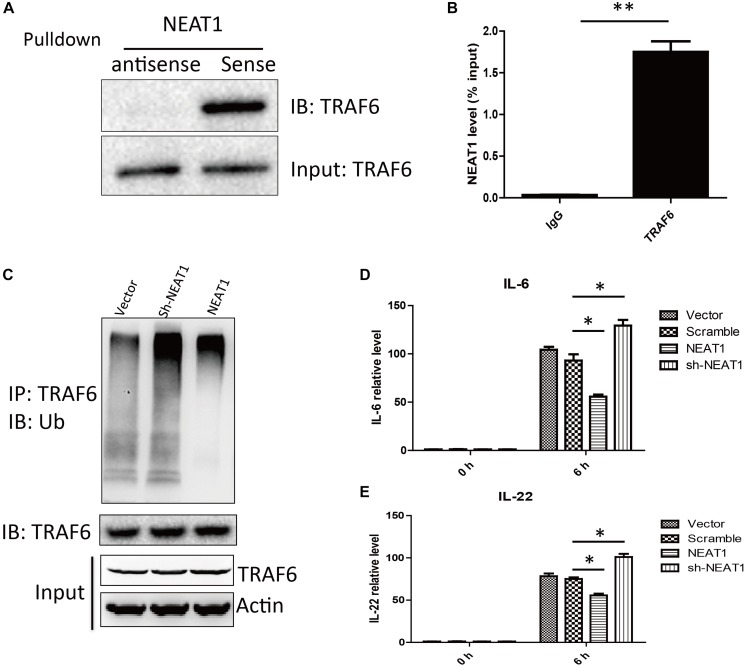
NEAT1 interacted with TRAF6 and repressed LPS induced inflammation response. **(A)** Western blot analysis of proteins bound to biotinylated NEAT1 in HepG2. **(B)** The recovery of NEAT1 is determined by immunoprecipitation with TRAF6 antibody in HepG2 cells. IgG served as negative control. Data is represented as mean ± SEM of three experiments. Two-tailed unpaired *t*-test is performed, and ^∗∗^ means *p* < 0.01 vs. IgG group. **(C)** Western blot analysis of the ubiquitination level of TRAF6 in HepG2. **(D,E)** qRT–PCR is used to study the effects of NEAT1 overexpression or NEAT1 knockdown on expression of IL-6 and IL-22 upon LPS stimulation in cultured HepG2. Two-tailed unpaired *t*-test is performed. ^∗^ means *p* < 0.05.

To search for NEAT1 interaction proteins in HepG2 cells, we labeled the lncRNA NEAT1 with biotin and performed RNA pull down assay, followed by mass spectrometry. TRAF6 was identified as a promising NEAT1 interaction protein. As TRAF6 played important roles in orchestrating the NF-κB and MAPK signaling pathways, we performed an independent RNA pull down and western blot to confirm the interaction between NEAT1 and TRAF6 (Figure 3A). Furthermore, an RNA immunoprecipitation experiment was carried out to verify the specific interaction (Figure 3B). NEAT1 may be a negative regulator of NF-κB and MAPK pathway through interacting with TRAF6. Auto-ubiquitination of TRAF6 is an essential post-translational modification for signal transduction. We wondered whether the interaction between NEAT1 and TRAF6 affected its ubiquitination. HepG2 cells were transfected with siNEAT1 and NEAT1, and the cell lysate was used for immunoprecipitation experiment with an anti-TRAF6 antibody and immunobloted with the anti-Ubiquitin antibody. These data demonstrated that NEAT1 significantly affected the ubiquitination level of TRAF6 (Figure 3C). Moreover, exogenous NEAT1 showed an anti-inflammation effect as the expressions of IL-6 and IL-22 decreased at 6 h post LPS stimulation, whereas NEAT1 knockdown showed an opposite effect (Figures 3D,E).

### NEAT1 Correlates With IL-6 and IL-22 Expression in ACLF Patients

**FIGURE 4:**
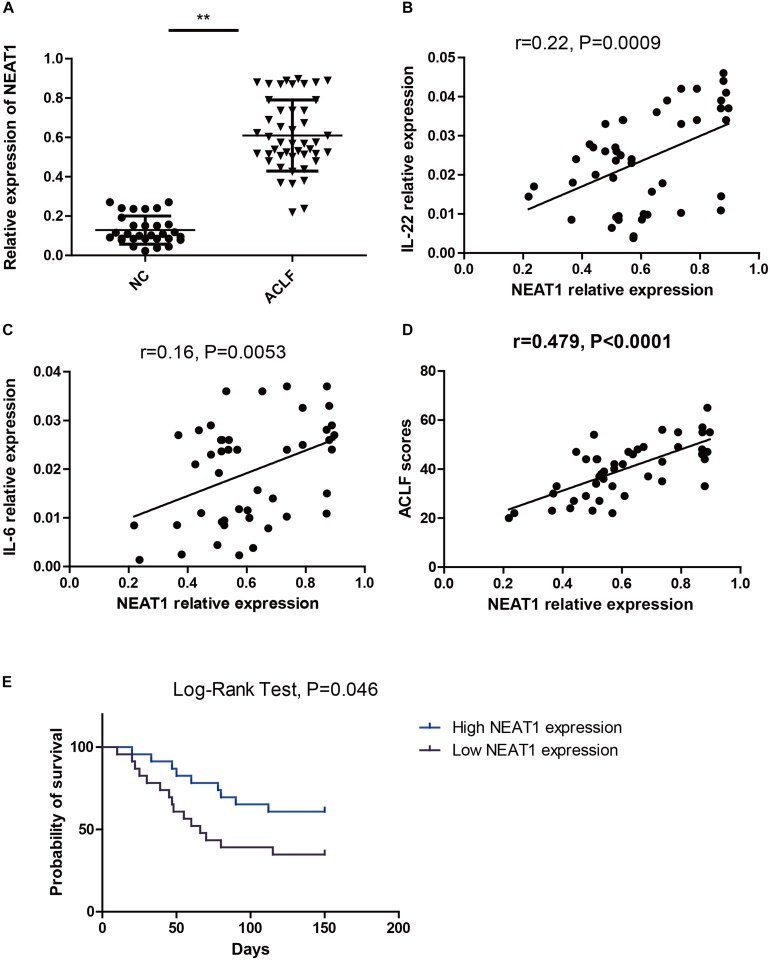
NEAT1 correlates with IL-6 and IL-22 expression in ACLF patients. **(A)** qRT–PCR is used to study the expression of NEAT1 in PBMCs from healthy controls (*n* = 30) and ACLF patients (*n* = 46) ^∗∗^ means *p* < 0.01. **(B,C)** The correlation between NEAT1 and IL-22 **(B)** IL-6 **(C)** in PBMCs from healthy controls (*n* = 30) or ACLF patients (*n* = 46) is performed by non-parametric correlation analysis method. **(D)** The correlation between NEAT1 and CLIF-C ACLFs on 28 day. **(E)** Expression of NEAT1 in predicting 150-day mortality rate.

Thirty healthy controls and forty six ACLF patients were enrolled in this study. The demographic and clinical characteristics were listed in Table 1. The expression levels of NEAT1 in PBMCs from ACLF patients and the healthy controls were evaluated. As shown in Figure 4A, ACLF patients had higher NEAT1 expression level than healthy controls. Since NEAT1 could regulate LPS stimulated inflammation genes, we wondered whether NEAT1 correlated with cytokines expression in ACLF patients. As shown in Figures 4B,C, NEAT1 positively correlated with IL-6 or IL-22 in ACLF patients. We also found that NEAT1 correlated with the ACLF disease activity (Figure 4D). High expression of NEAT1 is associated with poor prognosis of patients with ACLF (Figure 4E).

### NEAT1 Attenuated Liver Damage in ACLF Rat Model

**FIGURE 5:**
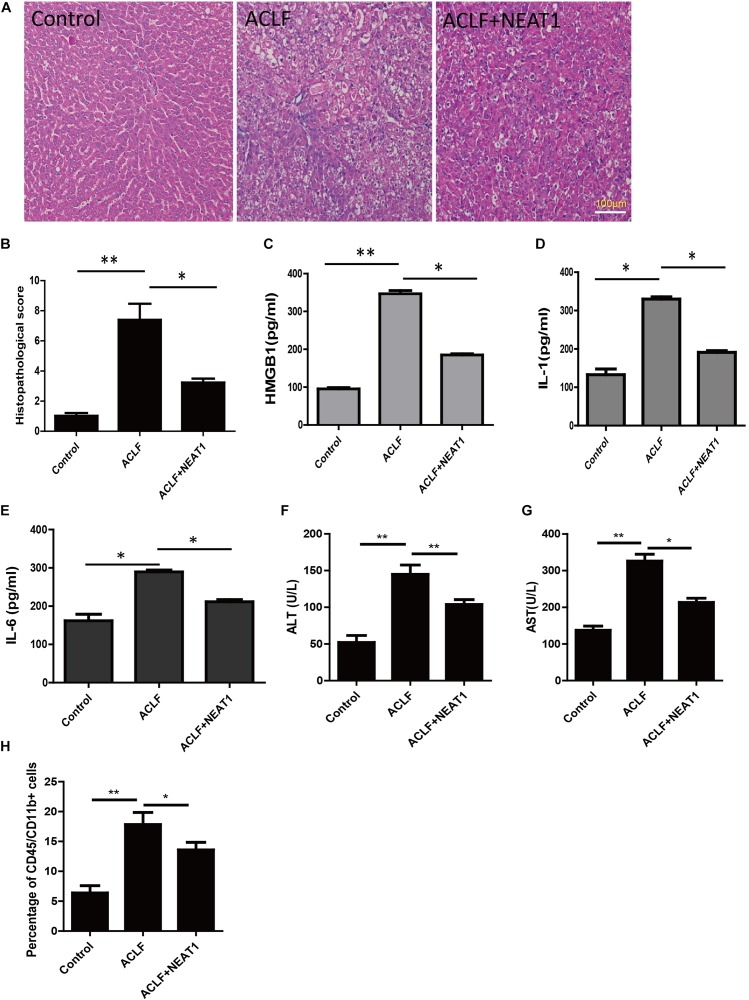
NEAT1 attenuated liver damage in ACLF rat model. **(A)** Representative pathological section of the liver from the control group (*n* = 6), ACLF group (*n* = 6), and ACLF + NEAT1 treatment group (*n* = 6). The ACLF liver has plenty of lesions, whereas there are significant reduced lesions in the ACLF + NEAT1 group **(B)** Histopathological score. **(C–E)** The levels of HMGB1 **(C)**, IL-1 **(D)**, IL-22 **(E)** in serum of these rats were detected. The respective cytokines were detected using ELISA. **(F,G)** Liver damage assay of ALT and AST. **(H)** Percentage of CD45/CD11b^+^ cells. ^∗^ means *p* < 0.05 and ^∗∗^ means *p* < 0.01

Acute-on-chronic liver failure was induced in rats as described in the methods. The function of NEAT1 was investigated in rats with ACLF induced by LPS and Gal-N challenge. The typical feature of ACLF was organ injury and liver failure. The representative pathological section was shown in the Figure 5A. The ACLF rats presented obvious liver inflammation and hemorrhaging compared to the normal control, whereas when administrated with NEAT1 adenovirus via the tail vein the mice showed very few liver lesions, with a lower liver injury score (Figure 5B). Liver damage was significantly attenuated upon NEAT1 treatment (*p* < 0.01). Moreover, plasma cytokines of IL-22, IL-6, and HMGB1 were significantly reduced in the NEAT1 adenovirus treated mice (Figures 5C–E). Substantial liver damage in ACLF rats was significantly weakened with NEAT1 treatment (Figures 5F,G). The percentage of CD45^+^/CD11b^+^ macrophages was increased in ACLF rats, and attenuated by NEAT1 administration (Figure 5H). Collectively, the data demonstrated that NEAT1 could effectively relieve liver dysfunction in ACLF rats.

## Discussion

A wealth of research has illustrated that lncRNAs are important regulators in inflammatory processes (Carpenter et al., 2013; Ilott et al., 2014; Liu et al., 2015). In our previous study, HepG2 cells were treated with LPS, and then the whole transcriptome sequencing is carried out to investigate the differential expressed lncRNA profiles. It is found that LPS can up-regulate lncRNA NEAT1 expression. Considering LPS stimulation can up-regulate NEAT1 expression, we wondered whether the amount of NEAT1 could regulate numerous inflammatory factors, including IL-6 and IL-22.

The expression of NEAT1 increased upon the activation of p38 and STAT1 pathways. NEAT1 could interact with TRAF6 and inhibit the ubiquitination of TRAF6, and finally weaken the LPS stimulated inflammation responses. The expression of NEAT1 increased obviously and peaked at 6 h after LPS treatment. In HepG2 cells, over-expression of NEAT1 could suppress inflammatory effects significantly.

Inflammation occurs in response to harmful stimuli, but the systemic inflammation responses during ACLF process is catastrophic (Cazzaniga et al., 2009). This concept is in consist with out previous findings that the use of TNF antagonists (TNFR:IgG-Fc) can alleviate liver injury (Xu et al., 2013). In this present study, NEAT1 over-expression down-regulated the level of IL-6 and alleviated the liver injury. Moreover, in ACLF animal model, NEAT1 adenovirus relieved organ dysfunction of these rats.

However, the protective function of NEAT1 may be cell type dependent. In HeLa cells, NEAT1 can promote the binding of transcription factor to IL-8 promoter and regulate IL-8’s expression (Imamura et al., 2014). In HepG2 cells, the expression of IL-8 seems not affected by NEAT1. The function mechanism of NEAT1 in different cell types needs to be deeply studied. LPS can activate NF-kB and MAPK pathways. NEAT1 was initially up-regulated by LPS treatment, and interacted with TRAF6 to further regulate the downstream signaling pathways. Ubiquitination of TRAF6 activates a complex containing MAP3K7/TAK1, TAB1, and TAB2 (Ninomiya-Tsuji et al., 1999; Takaesu et al., 2000). The activated complex then induces phosphorylation of the IKK (inhibitor of IκB kinase) complex, resulting in activation of NFKB (nuclear factor kappa-light-chain-enhancer of activated B cells) and induction of expression of genes encoding inflammatory cytokines. But the thoroughly regulation mechanism needs to be further investigated. NEAT1 may be a promising therapeutic target in the future.

Taken together, our study makes a connection between lncRNA and ubiquitin modification in inflammatory response. NEAT1 is profiled as a LPS stimulated lncRNA. Then the NEAT1 functions as a negative regulator in the inflammation process through interacting with TRAF6 and affecting its ubiquitination. Moreover, the expression of NEAT1 increases in PBMCs from ACLF patients compared to healthy controls. The NEAT1 will be a promising diagnosis biomarker for ACLF and indicator for disease pathogenesis in the future.

## Data Availability Statement

The raw data supporting the conclusions of this article will be made available by the authors, without undue reservation, to any qualified researcher.

## Author Contributions

QX, HW, and RH designed, coordinated the study, took responsibility for the integrity of the data and the accuracy of the data analysis, involved in administration, technical and material support, and study supervision. YX, ZC, YD, ZL, XX, RL, ZS, YL, and WC performed the experiments. YX, ZC, and YD drafted the manuscript. QX, HW, and YX obtained the funding. All authors critically revised the manuscript for important intellectual content, read and approved its final version.

## Conflict of Interest

The authors declare that the research was conducted in the absence of any commercial or financial relationships that could be construed as a potential conflict of interest. The reviewer HL declared a shared affiliation, with no collaboration, with several of the authors, YX, ZC, YD, ZL, XX, RL, ZS, YL, WC, HW, QX, to the handling Editor at the time of review.

## Footnotes

1 http://www.aaalac.org
