# Supplementary figures and images for: Long Non-coding RNA NEAT1 Alleviates Acute-on-Chronic Liver Failure Through Blocking TRAF6 Mediated Inflammatory Response
Source: Front Physiol. 2019 Dec 12;10:1503. doi: 10.3389/fphys.2019.01503 (PMC6920254; doi:10.3389/fphys.2019.01503)

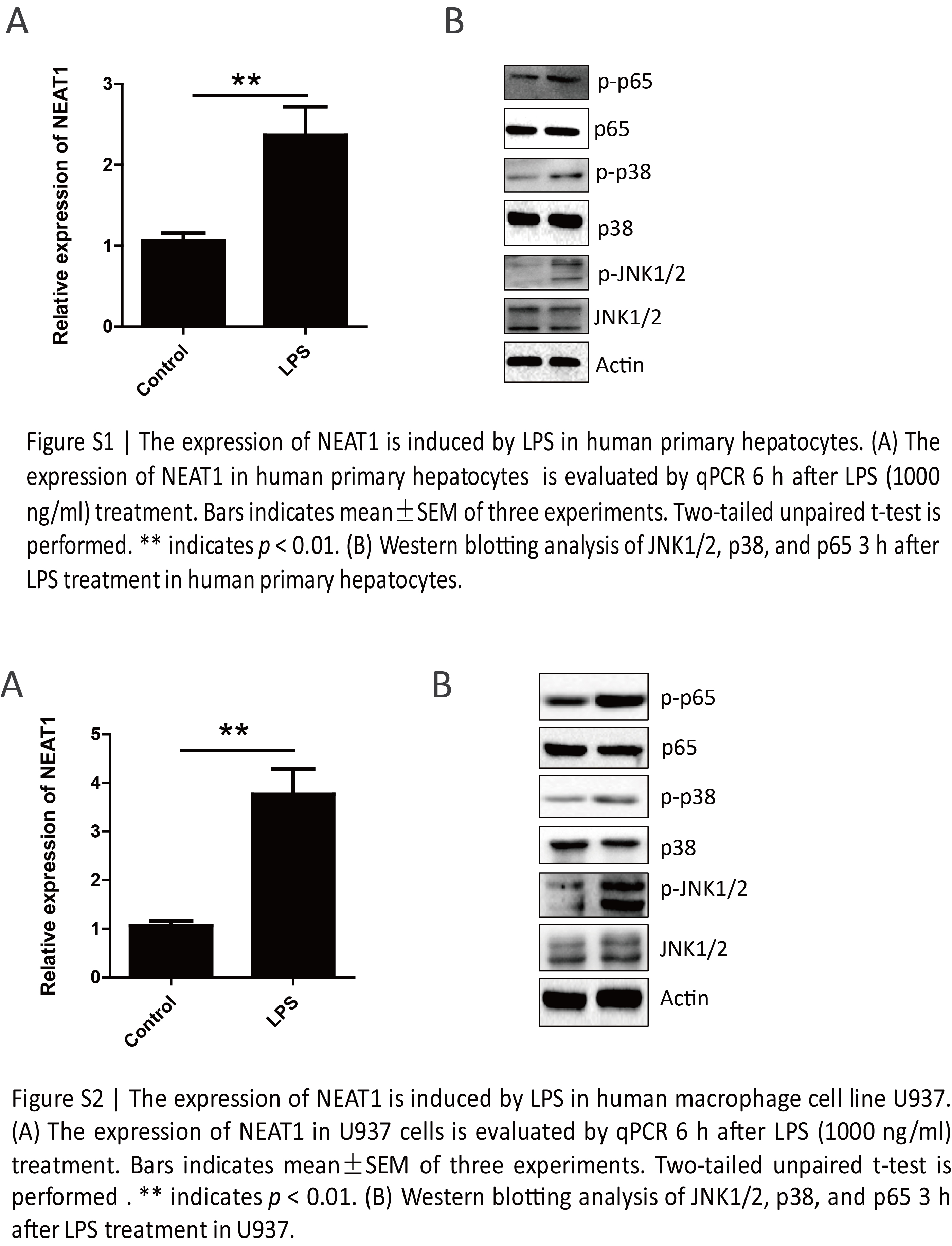

Supplement: Supplementary file 1 [file Image_1.jpg]
